# Functional Analysis of a Novel *HNF4A* Variant Identified in a Patient With MODY1

**DOI:** 10.1210/jendso/bvae090

**Published:** 2024-05-07

**Authors:** Shuntaro Morikawa, Hui Ling Ko, Ee Chee Ren, Kazuya Hara, Naoya Kaneko, Nozomi Hishimura, Akie Nakamura, Atsushi Manabe

**Affiliations:** Department of Pediatrics, Hokkaido University Hospital, Sapporo, 060-8648, Japan; Singapore Immunology Network (SIgN), Agency for Science, Technology and Research (A*STAR), Singapore 138648, Singapore; Singapore Immunology Network (SIgN), Agency for Science, Technology and Research (A*STAR), Singapore 138648, Singapore; Department of Microbiology and Immunology, Yong Loo Lin School of Medicine, National University of Singapore, Singapore 117597, Singapore; Department of Pediatrics, Chitose City Hospital, Chitose, 066-0033, Japan; Department of Pediatrics, Hokkaido University Hospital, Sapporo, 060-8648, Japan; Department of Pediatrics, Hokkaido University Hospital, Sapporo, 060-8648, Japan; Department of Pediatrics, Hokkaido University Hospital, Sapporo, 060-8648, Japan; Department of Pediatrics, Hokkaido University Hospital, Sapporo, 060-8648, Japan

**Keywords:** HNF4A-MODY, MODY1, HNF4α, diabetes, isoform, transcription

## Abstract

**Context:**

HNF4A–maturity-onset diabetes of the young (MODY1) is a relatively rare subtype of monogenic diabetes caused by loss of function of the *HNF4A* gene, which encodes the transcription factor HNF4α. HNF4α is known to form heterodimers, and the various combinations of isoforms that make up these heterodimers have been reported to result in a diversity of targeted genes. However, the function of individual HNF4α variant isoforms and the heterodimers comprising both wild-type (WT) and variant HNF4α have not yet been assessed.

**Objective:**

In this study, we analyzed the functional consequence of the *HNF4A* D248Y variant in vitro.

**Methods:**

We investigated the case of a 12-year-old Japanese girl who developed diabetes at age 11 years. Genetic sequencing detected a novel heterozygous missense *HNF4A* variant (c.742G > T, p.Asp248Tyr; referred as “D248Y”) in the patient and her relatives who presented with diabetes.

**Results:**

Although the WT HNF4α isoforms (HNF4α2, HNF4α3, HNF4α8, HNF4α9) enhanced the *INS* gene promoter activity in HepG2 cells, the promoter activity of D248Y was consistently low across all isoforms. The presence of D248Y in homodimers and heterodimers, comprising either HNF4α8 or HNF4α3 or a combination of both isoforms, also reduced the *INS* promoter activity in Panc-1 cells.

**Conclusion:**

We report the clinical course of a patient with HNF4A-MODY and the functional analysis of novel *HNF4A* variants, with a focus on the isoforms and heterodimers they form. Our results serve to improve the understanding of the dominant-negative effects of pathogenic *HNF4A* variants.

Maturity-onset diabetes of the young (MODY) is characterized by early onset of diabetes before age 25 years, absence of β-cell autoimmunity, sustained pancreatic β-cell function, nonobesity, and an autosomal dominant inheritance [1]. At least 14 types of MODY have been reported, among which HNF4A-MODY (MODY1) accounts for 3.8% of cases in the Japanese pediatric MODY population [2]. The *HNF4A* gene (*HNF4A*) is the causative gene for HNF4A-MODY and encodes the transcription factor HNF4α, which is expressed dominantly in the liver and pancreas, and is essential for glucose transport and metabolism [1]. Herein, we describe the clinical course of a patient with HNF4A-MODY and assess the function of this novel *HNF4A* variant, thereby contributing to the list of clinically pathogenic *HNF4A* variants associated with HNF4A-MODY.

## Materials and Methods

### Study Participants

Informed consent was obtained from the patient's parents on her behalf. This study was approved by the ethics committee of Hokkaido University Hospital (approval No. 022-0291).

### Sequence Analysis

Genomic DNA was extracted from the peripheral blood leukocytes or saliva. Targeted next-generation sequencing for the proband was performed by the Kazusa DNA Research Institute using the gene panel for “glucometabolic disorders,” comprising 18 genes (*ABCC8*, *GATA6*, *GCK*, *GLUD1*, *HNF1A*, *HNF1B*, *HNF4A*, *INS*, *INSR*, *KCNJ11*, *NEUROD1*, *PDX1*, *AIRE*, *FOXP3*, *HADH*, *KLF11*, *WFS1*, *PIK3R1*). The detected variant was confirmed using polymerase chain reaction (PCR)-direct sequencing at the Department of Pediatrics, Hokkaido University Hospital. Sequence analysis of *HNF4A* for the patient's father, mother, paternal grandmother, and uncle was performed using PCR-direct sequencing.

### Plasmids

Myc-tagged human *HNF4A2* expression plasmids were purchased from Addgene (FR_HNF4A2, plasmid No. 31100) to determine the relative expression of HNF4α in the nucleus. For luciferase assays, untagged HNF4α overexpression constructs were generated as described previously [3]. Site-directed mutagenesis was performed using the PrimeSTAR Mutagenesis Kit (Takara, catalog No. R046A) to create the *HNF4A* variant (c.742G > T, p.Asp248Tyr). Trans-IT LT1 (catalog No. MIR2300; Mirus) was used for all plasmid transfections. The transfected cells were collected 48 hours after transfection.

*INS* luciferase reporter constructs were generated by inserting the human insulin gene promoter sequence (−500 bp upstream of the transcription start site) into the PGL3 Basic promoter through *KpnI* and *XhoI* restriction sites.

### Cell Culture

HepG2 cells were provided by Dr Hiraku Kameda (Department of Endocrinology, Hokkaido University, Japan). Panc-1 cells were purchased from the American Type Culture Collection. The cells were cultured in Dulbecco’s modified Eagle’s medium (1.0 g/L glucose) (Nacalai Tesque, catalog No. 08456-36) supplemented with 10% fetal bovine serum (Thermo Fisher Scientific, catalog No. 10099141) and 100 U/mL penicillin-streptomycin (Thermo Fisher Scientific, catalog No. 15140122).

### Luciferase Assay

For the luciferase assay, 2 × 10^5^ HepG2 or Panc-1 cells were seeded in triplicate into clear-bottom black well plates and transfected with 50 ng of *HNF4A* overexpression constructs or empty vector, together with 200 ng of *INS* promoter reporter construct using 0.22 µL of Lipofectamine 2000 (Thermo Fisher Scientific, catalog No. 11668027). Forty-eight hours later, the cells were lysed at 37 °C for 30 minutes, with 50 µL reconstituted Passive Lysis 5× Buffer (Promega, catalog No. E1941). Finally, luminescence was acquired on the Promega GloMax plate reader under recommended conditions using 50 µL of Luciferase Assay System substrate (Promega, catalog No. E1500).

### Immunoblot

Cells were washed in cold phosphate-buffered saline (PBS) and immediately lysed with NE-PER Nuclear and Cytoplasmic Extraction Reagents (Thermo Fisher Scientific, catalog No. 78833) containing Protease Inhibitor Cocktail (Merck, catalog No. P8340) and 10 mM phenylmethylsulfonyl fluoride. Next, 8 mg of nuclear lysates were boiled at 95 °C for 15 minutes in 2× Laemmli Sample Buffer (Bio-Rad, catalog No. 1610737) containing 10% β-mercaptoethanol. The proteins were resolved by sodium dodecyl sulfate–polyacrylamide gel electrophoresis and transferred to Immobilon-P^SQ^ PVDF membrane (Merck, 0.2-µm pore size, catalog No. ISEQ85R). The primary antibodies used for immunoblotting were anti-fibrillarin (1:1000, Abcam, catalog No. ab5821, RRID:AB_2105785) and anti-HNF4α (1:1000, Thermo Fisher Scientific, catalog No. MA1-99, RRID:AB_2633309 or catalog No. 417800, RRID:AB_2532198), whereas the secondary antibodies were anti-mouse immunoglobulin G (IgG) horseradish peroxidase–linked antibody (1:3000, CST, catalog No. 7076, RRID:AB_330924) and anti-rabbit IgG horseradish peroxidase–linked antibody (1:3000, CST, catalog No. 7074, RRID:AB_2099233). Bands were detected by SuperSignal West Femto Maximum Sensitivity Substrate (Thermo Fisher Scientific, catalog No. 34095) diluted 3-fold in water, and images were acquired using ChemiDoc MP (Bio-Rad).

### Immunofluorescence Staining

HepG2 cells were seeded and cultured on Nunc Lab-Tek II Chamber Slides (Thermo Fisher Scientific, catalog No. 154461PK) for 24 hours prior to transfection with *HNF4A2* overexpression plasmids (2.5 µg per well). After 48 hours, the cells were incubated with 4% paraformaldehyde for 10 minutes and then permeabilized with 0.1% Triton X-100 in PBS for 10 minutes at room temperature. Blocking was performed by treating the cells with 2% bovine serum albumin in PBS-Tween for 1 hour. Anti-Myc-Tag (9B11) antibody (CST, catalog No. 2276, RRID: AB_ 331783) was used as the primary antibody at a 1:500 dilution, and Alexa Fluor 594 donkey anti-mouse IgG (Invitrogen, catalog No. A11037, RRID:AB_2534095) in 1:200 was used as the secondary antibody. The cells were observed using a FluoView 1000D microscope (Olympus).

### Quantitative Real-Time Polymerase Chain Reaction

Total RNA was extracted from HepG2 cells using the RNeasy Mini Kit (Qiagen, catalog No. 74106) and reverse-transcribed using High-Capacity complementary DNA Reverse Transcription Kits (Thermo Fisher Scientific, catalog No. 4368814). The quantitative PCR was performed with StepOnePlus (Applied Biosystems) and PowerUp SYBR Green Master Mix (Thermo Fisher Scientific, catalog No. A25742). All reactions were performed in triplicate and the experiments were repeated 2 times. The quantitative PCR primer sequences used in this study are as follows: *APOA4* forward, CACGGTGATGTGGGACTACTTC; *APOA4* reverse, CCAAGTTTGTCCTGGAAGAGGG; *G6PC1* forward, GCTGTGATTGGAGACTGGCTCA; *G6PC1* reverse, GTCCAGTCTCACAGGTTACAGG.

## Results

### Clinical Course of a Patient With HNF4A-MODY

**Figure 1.**
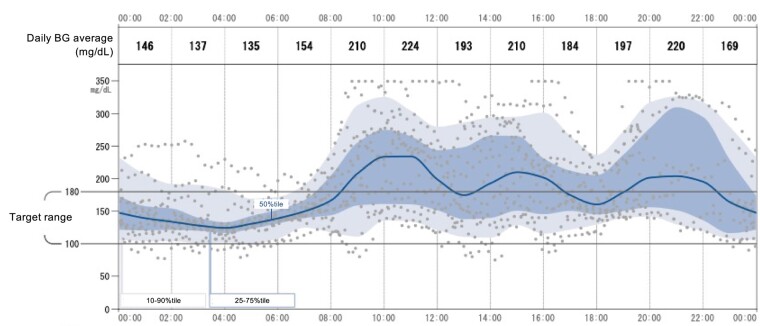
Results of continuous glucose monitoring before insulin initiation. For the most part, the median blood glucose (BG) during the day was higher than the target range.

An 11-year-old Japanese girl was diagnosed with glycosuria through a school urine screening program. Hyperglycemia was noticed at a local clinic when she was aged 12 years; thereafter, she was referred to our hospital for further evaluation. She was born at 41 weeks of gestation, with a birth weight of 3382 g (+0.29 SD for a normal Japanese girl), and there was no episode of hyperinsulinemia in her medical history, including that of her neonatal period. On admission, her height was 144.0 cm (−0.89 SD for a normal Japanese girl), and her weight was 44.6 kg (body mass index: 21.5, 84.7th percentile). Her menarche was at age 10 years, and her breast was Tanner stage 3 at the time of admission. Laboratory examination revealed hyperglycemia (426 mg/dL: 23.6 nmol/L), elevated glycated hemoglobin A_1c_ (12.7%), and positive urine glucose and ketones. Antiglutamic acid decarboxylase, anti-insulin, and anti-insulinoma–associated antigen-2 antibodies were negative. The homeostasis model assessment insulin resistance (HOMA-IR) index, as a marker of insulin resistance, was 3.9 (normal range <1.6), and the HOMA-β index, as a marker for insulin-secreting capacity, was 13 (normal range, 40-60), indicating insulin resistance and impaired insulin secretion. Continuous glucose monitoring revealed hyperglycemia during the daytime (Fig. 1). The glucagon tolerance test, a test commonly performed to assess endogenous insulin secretion capacity [4], revealed peak levels of blood glucose and C-peptide immunoreactivity (CPR) of 177 mg/dL (9.82 mmol/L) and 2.8 ng/mL (normal range, 5.0-7.0 ng/mL), respectively, suggesting insufficient insulin secretion (Table 1).

**Table 1.** Glucagon tolerance test of the patient on admission

Glucagon tolerance test		
	Basal	Peak
Blood glucose, mg/dL	153	177
CPR*^a^*, ng/mL	1.5	2.8

Abbreviation: CPR, C-peptide immunoreactivity.

^*a*^The normal range for peak CPR after glucagon administration is 5.0 to 7.0 ng/mL.

### Identification of a Novel *HNF4A* Variant

**Figure 2.**
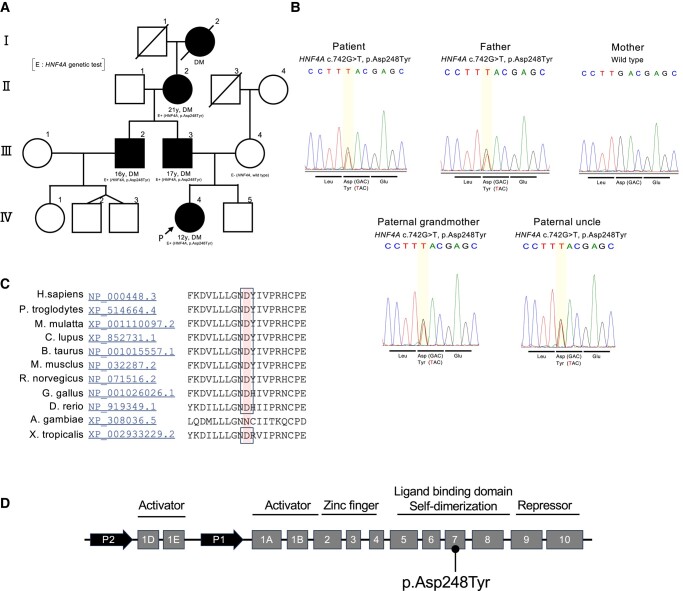
Characterization of the novel D248Y-HNF4α variant. A, Pedigree of the patient's family. The patient (P, IV-4), her father (III-3), paternal grandmother (II-2), uncle (III-2), and great grandmother (I-2) developed diabetes (DM) at the indicated age. The onset of DM in the patient's paternal great-grandmother (I-2) was unknown. The patient's mother (III-4) does not have DM. B, Sanger sequencing chromatograms. The heterozygous missense variant in *HNF4A* (c.742G > T, p.Asp248Tyr) was detected in the patient, her father, paternal grandmother, and uncle. C, Amino acid sequence of HNF4α protein. Residue D248 is well conserved across vertebrae species. Among the aligned sequences, the sequence of *A gambiae* is least conserved as it is derived from an invertebrate. The sequence data were obtained from HomoloGene, accessed October 17, 2023 (https://www.ncbi.nlm.nih.gov/homologene/). D, Schematic representation of *HNF4A* exons. The black dot represents the variant identified in this study.

The patient was the second child in her family, and her father, paternal grandmother, uncle, and great-grandmother had all developed diabetes (Fig. 2A). Genetic testing revealed that the patient was heterozygous for a novel missense variant in *HNF4A* (c.742G > T, p.Asp248Tyr; referred to as “D248Y”) (Fig. 2B), which is not registered in ClinVar, gnomAD, or HGMD (as of November 1, 2023). Further genetic tests for her family confirmed that the D248Y variant was also carried by her living relatives who presented with diabetes (father, paternal grandmother, and uncle), but not by her mother who did not present with diabetes (Fig. 2B). Genetic testing could not be performed on the deceased great-grandmother. As HNF4α is a transcriptional regulator of the insulin gene [5], these findings suggest a causal relationship between D248Y and the presentation of diabetes in this family. Our hypothesis is supported by multiple in silico analyses for the variant, which agreed with the labeling of the variant as “damaging” for HNF4α functions (Table 2). Residue D248 was highly conserved across vertebrate species (Fig. 2C), yielding only sequence variation in invertebrates. D248 is located in exon 7 of *HNF4A*, corresponding to a region that encodes the ligand-binding and self-dimerization domains (Fig. 2D).

**Table 2.** In silico analysis of HNF4A (c.742G > T, p.Asp248Tyr)

Software	PolyPhen-2	M-CAP	PROVEAN	SIFT	MutationTaster
Outcome	Probably damaging	Damaging	Damaging	Damaging	Disease causing
Score	1	0.834	8.75	0.001	1

### *HNF4A* (D248Y) Is a Loss-of-Function Pathogenic Variant

**Figure 3.**
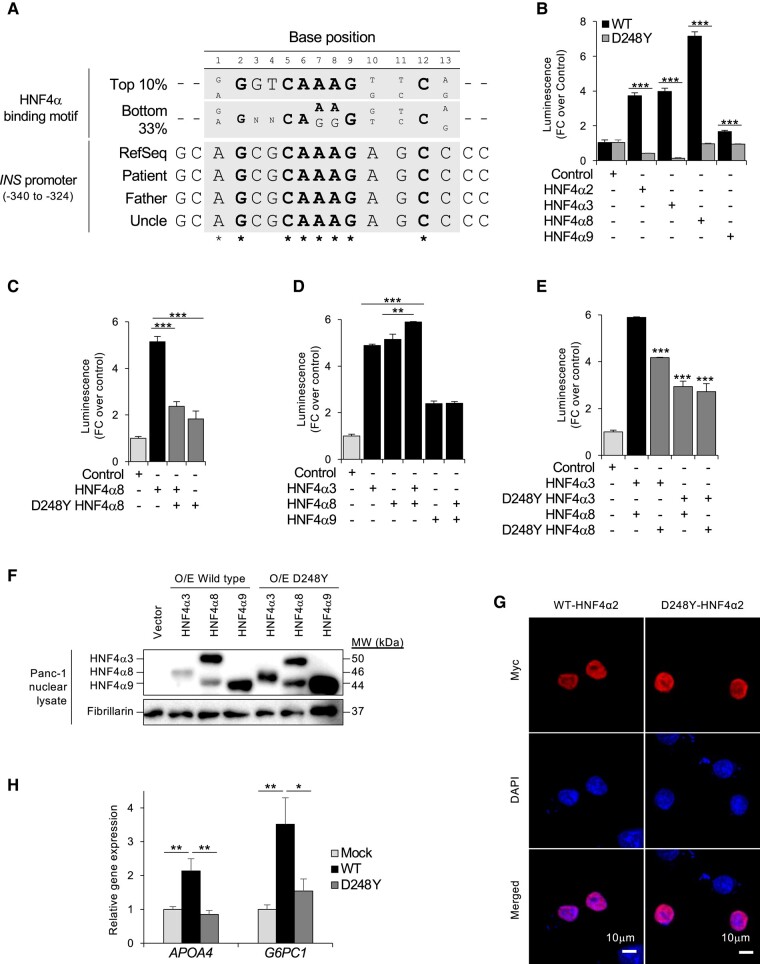
Functional characterization of the D248Y-HNF4α variant. A, The HNF4α binding motif (gray box) has been described previously [6], and is aligned with nucleotide position −340 to −324 of the *INS* reference sequence (RefSeq). The same can be observed in the patient and her family's *INS* sequence, which were obtained from Sanger sequencing. Bold nucleotides are conserved across strong and weak HNF4α binding sequences. B, Luciferase reporter assay for human insulin promoter in the presence of wild-type (WT) or variant HNF4α overexpressed in HepG2 cells. Data are shown as the mean fold-change ± SEM, using overexpressed empty plasmid control as reference. ***P less than .001. C to E, Luciferase reporter assay for human insulin promoter for combinations of overexpressed WT and variant HNF4α in Panc-1 cells. Data are shown as the mean ± SEM, relative to the luminescence of the overexpressed empty vector control. ***P less than .001; **P less than .01. F, Nuclear lysate immunoblot images of WT or D248Y-HNF4α in transfected Panc-1 cells. G, Typical immunofluorescent images of HepG2 cells transfected with Myc-tagged WT or D248Y-HNF4α. H, messenger RNA expression levels of *APOA4* and *G6PC1* normalized to *18srRNA* in HepG2 cells overexpressing WT or D248Y-HNF4α (n = 6). Data are shown as the mean ± SEM, *P less than .05; **P less than .005.

As HNF4α functions as a dimer of its isoforms to regulate gene expression, we speculated that mutations in this domain would affect transcription efficiency of *INS*, contributing to aberrant insulin expression. To substantiate this, we analyzed the human *INS* promoter and found a canonical HNF4α binding site [6] at 326 to 338 nucleotides upstream of the transcription start site (Fig. 3A). This 13-nucleotide sequence and flanking ±2 nucleotides were not mutated in the patient, father, and uncle's *INS* promoters, suggesting that any changes in *INS* activity is a direct consequence of altered HNF4α function. To determine whether D248Y affects insulin expression, we compared how wild-type (WT) and D248Y overexpression affected transcription of the human insulin promoter (P*_INS_*). A promoter reporter assay was performed on HepG2 cells using a luciferase reporter construct driven by P*_INS_* (−500 bp from the transcription start site). As the most highly expressed isoforms in the pancreas are HNF4α3, HNF4α8, and HNF4α9 [3], all 3 were tested for relative capacity to drive transcription in comparison to HNF4α2, which is highly expressed in liver cells and acts as a control. All isoforms showed increased reporter luminescence compared to the empty vector control (Fig. 3B), with HNF4α8 producing the greatest luminescence, followed by HNF4α3 and HNF4α2. HNF4α9 was the weakest in activating P*_INS_*. With D248Y, all 4 isoforms produced a marked loss in luminescence, reaching levels comparable to or below those of the empty vector control. This suggested that D248Y is a loss-of-function pathogenic variant that ablates transcription.

To verify the results, the experiment was repeated in Panc-1 pancreatic cells for HNF4α8. Consistent with the HepG2 data, overexpressed HNF4α8 increased transcription at the insulin promoter (Fig. 3C). When half of the amount of construct was replaced with D248Y-HNF4α8 to recapitulate the heterozygous state, P*_INS_* activity was significantly reduced by more than 50%. Interestingly, no further loss in promoter activity was observed when all overexpression constructs were replaced with D248Y-HNF4α8, suggesting that the presence of D248Y-HNF4α8 alone was sufficient to disrupt transcription at the P*_INS_*.

Next, we tested whether pancreatic isoform heterodimers were similarly affected by the D248Y variant. Consistent with the HepG2 data, P*_INS_* in Panc-1 cells was strongly activated by HNF4α3 and HNF4α8 but not HNF4α9 (Fig. 3D). Luminescence was further enhanced when both isoforms were concurrently expressed, suggesting that HNF4α3-8 is the most potent P*_INS_* activator in Panc-1 cells. In contrast, HNF4α8-9 activated P*_INS_* to a similar extent as HNF4α9. When the D248Y variant was introduced singly into the monomeric units of HNF4α3-8, P*_INS_* activity was markedly reduced (Fig. 3E). Transcription was not further reduced when both monomeric units of HNF4α3-8 were mutated. As the variant was well expressed (Fig. 3F) and did not differ from WT HNF4α in terms of nuclear localization (Fig. 3G), the marked loss of P*_INS_* activities was attributed to the loss of HNF4α function. Taken together, these data suggest that the D248Y variation exerts a dominant effect on HNF4α-dependent transcription in an isoform-independent manner to disrupt insulin expression.

To confirm that D248Y was a loss-of-function mutant, we assessed the expression of other HNF4α target genes in HepG2 [7, 8]. Consistent with our hypothesis, *APOA4* and *G6PC1* expression were specifically upregulated in HepG2 cells in the presence of overexpressed WT *HNF4A2* and was significantly reduced by overexpression of D248Y (Fig. 3G). Thus, D248Y is a loss-of-function variant that abrogates HNF4α function as a transcription activator at multiple HNF4α target gene loci.

## Discussion

Here, we describe a Japanese patient with MODY with a heterozygous *HNF4A* variant (c.742G > T, p.Asp248Tyr) inherited in an autosomal-dominant manner from her father. Further genetic tests in the family tree confirmed the autosomal-dominant inheritance of the variant, which was passed down from her paternal grandmother with diabetes to both her sons. According to the guidelines of the American College of Medical Genetics and Genomics [9], the D248Y variant is deemed “pathogenic”. The D248Y variant has not been documented previously in multiple established databases (PM2), and extensive computational analyses have indicated that the variant is pathogenic (PP3). In this study, we provide conclusive functional evidence for the deleterious effects of the D248Y variant on HNF4α target gene expression (PS3). The D248Y variant contributed to the patient's condition, leading to a diagnosis of HNF4A-MODY (MODY1). The clinical course of the other family members with the D248Y variant was also consistent with HNF4A-MODY. While some *HNF4A* variants are known to cause congenital hyperinsulinism in the neonatal period, the patient was not overweight at birth nor hypoglycemic, suggesting insulin hypersecretion. Additionally, the patient did not develop lipid abnormalities, a complication often seen in patients with HNF4A-MODY [1, 10].

Twelve HNF4α isoforms have been identified in humans to date [3]. To better understand the context of the mutation with respect to the patient's loss of insulin secretion, we compared how WT and variant pancreatic HNF4α isoforms affect P*_INS_* transcription activity in luciferase reporter assays. Our data from pancreatic and liver cell lines demonstrate that D248Y is a dominant mutant that disrupts transcription at P*_INS_*. The effect of the D248Y variant was not restricted to P*_INS_*, as the HNF4α target genes *APOA4* and *G6PC1* were also significantly downregulated in the presence of the variant. Thus, the mutation in the ligand-binding and dimerization domain abrogates HNF4α function as transcription factor. Although several studies have analyzed the function of variant HNF4A in vitro [11, 12], this is the first study to examine the transcriptional activity of variant HNF4α on an isoform and heterodimer basis.

## Data Availability

Data sharing is not applicable to this article as no data sets were generated or analyzed during the current study.

## Abbreviations

CPR: C-peptide immunoreactivity
MODY1: maturity-onset diabetes of the young
PBS: phosphate-buffered saline
PCR: polymerase chain reaction
P*_INS_*: human insulin promoter
WT: wild-type
